# Manipulation of zebrafish’s orientation using artificial cilia in a microchannel with actively adaptive wall design

**DOI:** 10.1038/srep36385

**Published:** 2016-11-08

**Authors:** Karthick Mani, Tsung-Chun Chang Chien, Bivas Panigrahi, Chia-Yuan Chen

**Affiliations:** 1 Department of Mechanical Engineering, National Cheng Kung University, Tainan 701, Taiwan

## Abstract

The zebrafish is a powerful genetic model organism especially in the biomedical chapter for new drug discovery and development. The genetic toolbox which this vertebrate possesses opens a new window to investigate the etiology of human diseases with a high degree genetic similarity. Still, the requirements of laborious and time-consuming of contemporary zebrafish processing assays limit the procedure in carrying out such genetic screen at high throughput. Here, a zebrafish control scheme was initiated which includes the design and validation of a microfluidic platform to significantly increase the throughput and performance of zebrafish larvae manipulation using the concept of artificial cilia actuation. A moving wall design was integrated into this microfluidic platform first time in literature to accommodate zebrafish inside the microchannel from 1 day post-fertilization (dpf) to 6 dpf and can be further extended to 9 dpf for axial orientation control in a rotational range between 0 to 25 degrees at the minimum step of 2-degree increment in a stepwise manner. This moving wall feature was performed through the deflection of shape memory alloy wire embedded inside the microchannel controlled by the electrical waveforms with high accuracy.

The zebrafish made its first splash as an ideal model for genetic studies in vertebrate development due to its favorable biological characteristics such as high fecundity, transparency, short generation interval, and external fertilization^1^. Zebrafish genetics entered the picture and gained prominence with the large-scale mutagenesis screens performed for great potentials in genetic saturation screen^2^. From the time in the 1990 s and in the following years, zebrafish genetics was nearly exclusively confined to forward genetic studies, exploiting the huge number of available mutant strains, many of which proofed to be relevant to the understanding of pathogenesis of a number of human diseases^3^,^4^. In addition, compare to the human reference genome it shows that over 70% of human genes have at least one obvious zebrafish orthologue which evidences that zebrafish bears significantly genetic similarity to human^5^. Genetic studies of human disorders also benefit from the investigation of specific target genes of interest. Aside from that, National of Health has recognized zebrafish as the third animal model, and large numbers of zebrafish are available to facilitate studies related to gene functions and the identification of cellular targets of new compounds^6^. The trend in conducting zebrafish for human subjects is expected to be prospering for the decades to come.

Still, the lack of a trustworthy tool to manipulate zebrafish orientation during genetic screening hinders the practical realization of the full potential in modeling human diseases with zebrafish. To-date most of the zebrafish screening and imaging or related experiments rely heavily on experienced researchers with tediously and roughly manual operation for zebrafish orientation control to visualize the region of interest. This poses a significant barrier to batch-process large quantities of zebrafish which are highly required during the drug and gene processes. On top of that, zebrafish larvae are extremely fragile and vulnerable under the subjection of external forces such as the force applied from forceps during the manual processing of zebrafish, and it may cause detrimental impact to this animal model. As a remedy, microfluidic based manipulation platforms have sparked the interest through automated zebrafish processing. Several microfluidic platforms have been demonstrated to successfully facilitate zebrafish manipulation in more automatic and robust fashions. For example, an entrapment device that is agarose-free was reported to position zebrafish in a predictable array using pipettes. Addition access ports above the zebrafish are also available to administer local drug treatments^7^. A ZebraBeat device which integrated the imaging analysis function into a flexible platform is aimed to record cardiac activities of zebrafish in non-intrusive, low cost, and high reproducibility fashions^8^. Another study reported a method for zebrafish immobilization during imaging through fluorinated ethylene propylene tubes. In particular this refinement of zebrafish is also compatible for emerging optical imaging techniques^9^. Time-lapse brain activities of zebrafish is also applicable with a “Fish-Trap” array where the hydrodynamic force was employed to load, immobilize, and orient zebrafish larvae. This platform shows a promising alternative to exploit neurotoxin derivatives as therapeutic agents with zebrafish^10^. A toxicity test of zebrafish was reported to identify the effect of valproic acid (VPA) which may cause birth defects in children born to women taking this drug during pregnancy. This study was conducted by a multi-channel microfluidic perfusion platform to assess zebrafish responses which enabled drug treatment with multiple controlled dosages for an organ-level drug screen test^11^. A fully automated vertebrate screening platform that can orient zebrafish in a step-wised manner was announced which provides high-resolution and distortion-free imaging qualities of zebrafish larvae. A cross-correlation algorithm was employed to enable the auto-identification function in zebrafish positioning^12^. Aside from that, when it comes to the time-lapse imaging of zebrafish which can reveals important information on angiogenesis and vasculogenesis, the significantly morphological changes of zebrafish during early development post a notable barrier hindering the practical applications in such imaging procedures. To-date there is no valid platform available to accommodate such rapid growth of zebrafish. In short, the aforementioned methods shed light on better manipulation in zebrafish processing. To further extend the capability of microfluidic based platform in particular with a specific aim to improve the imaging quality and facilitate the time-lapse imaging, a new design is highly demanded from the perspective of orientation control of zebrafish during imaging.

Propelled by this motivation, an ideology was originated with an adaptive wall concept that can dynamically accommodate the dramatically morphological change of developing zebrafish inside a microchannel in an automated manner. Combined this feature with the inclusion of our previously reported artificial actuation for axial orientation control^13^, multiple anatomical viewing angles of zebrafish imaging over the entire early developing stages (from a fertilized egg to a larva) become feasible. The integration of these two important functions which can be actuated in synchronized manner is also advantageous for three-dimensional (3D) cardiovascular network reconstruction. Such post-processing results are envisaged that can improve the understanding of major arterial related disease such as arteriovenous malformations (AVMs) that is directly linked to pathological hemodynamics. On top of that, additions ports can be included in the microchannel network for the administration of drug treatment on a specific body part of the tested larval zebrafish which is beneficial especially in the animal test phase of new drug discovery and development. In this work a platform equips with an actively moving wall design is presented through the implementation of temperature sensitive Nickel Titanium wire to the artificial cilia based microchannel for repaid orientation control of zebrafish during time-lapse imaging. The technical development and validated tested results of the presented platform progress logically each immediately followed by pertinent discussion. The implications of presented platform in the context of *in vivo* imaging and control accuracy are presented further along with their limitations. Finally, in the Conclusion section, the utility of this work is summarized.

## Material and Methods

### Zebrafish lines and fluorescent microscopy

The wild type transgenic zebrafish lines Tg (gata1: DsRED), expressing red fluorescent protein (DsRED) signal from the blood cells were considered for this experiment. The specimens were raised in a re-circulating aquatic system (AZ- 303, GENDANIO, Taiwan) by following certified guidelines^6^,^14^. Furthermore, the zebrafish lines are being nurtured at a temperature of 28 °C in a well-regulated environment where vertebrate circadian rhythm (light, 14 h: dark, 10 h) has been constantly maintained. Zebrafish ranging from 1 days post-fertilization (d.p.f.) to 6 d.p.f. were considered for this experiment. The zebrafish embryos for the experiment were procured via random matting of the maintained zebrafish lines. Three minutes prior to the experiment, these zebrafish were anesthetized through Tricaine dosage of 0.154 ml/l, that was premediated by observing the behavioral response of zebrafish^13^. Subsequently, the zebrafish was introduced from petri dish to microchannel by generating suction force using the connected syringe. Only one zebrafish is measured at a time. Finally pulsating pressure is applied on the syringe’s plunger by gently tapping the end of the syringe’s plunger with a finger. This generates a pulsating flow inside the microchannel making the zebrafish travel across the microchannel in a series of small movements to avoid any possible damage to the zebrafish during transportation. In order to acquire fluorescent images, a microscope (BX60, Olympus Corp., Japan) equipped with a high-speed camera (NR4-S2, IDT, Tallahassee, FL, USA) having a frame rate of 2,000 Hz at a resolution of 1024 × 1024 pixels was employed. Moreover, all the experiments were conducted according to the approved guidelines issued by the *Institutional Animal Care* and *Use Committee* (IACUC) of National Cheng Kung University (Approval Number: 104103). Rigorous efforts were made to minimize the suffering of the animal model.

### Fabrication of the artificial cilia embedded microchannel with SMA fixture

**Figure 1:**
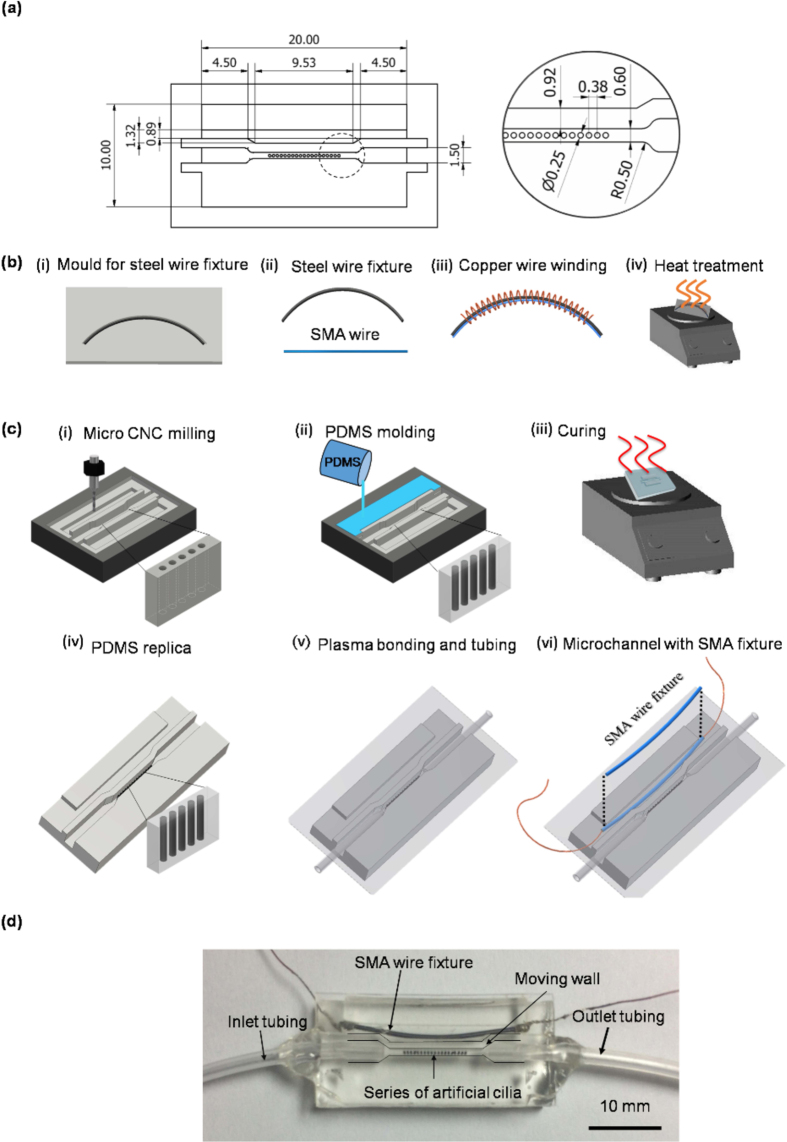
Schematic depiction of the microchannel with moving wall structure. (**a**) Dimensional details of the microchannel (all the units are expressed in mm). (**b**) Detailed fabrication of SMA wire fixture. (**c**) Fabrication process of the microchannel. (**d**) Top view of the photographed microchannel with an array of artificial cilia and SMA fixture.

In order to accommodate rapid morphological changes of zebrafish along with their orientation control, an artificial cilia based microchannel with moving wall structures were fabricated by means of rapid and reliable micro machining followed by elastomer casting. The dimensional details of the microchannel with moving wall is given in the Fig. 1(a). The moving wall structure of the microchannel was designed to be activated through the shape setting of shape memory alloy (SMA) wire fixture. The SMA wire fixture was designed in an arc shape through a molding step and straightened thereafter in the room temperature, supposed to regain its original arc shape with the application of heat by virtue of electrical energy, deemed to push the microchannel wall so that it could be able accommodate the rapid morphological changes of zebrafish. To fabricate the SMA wire fixture and towards attuning its shape setting several steps of micromachining, bending and heat treatment process were employed (Fig. 1b). Considering the design of the microchannel, the arc length of the SMA wire fixture was determined to be 20 mm. In order to provide the desired shape to the SMA wire fixture, 2 mm diameter steel wire was used as the base material that was priori casted on an aluminum mould of the desired arc shape. Subsequently, SMA wire was cut at the same length and fastened on the steel wire base through the help of 25-gauge copper wire. The whole fixture was then subjected to heat treatment process. For the heat treatment, the complete fixture was heated on a ceramic hot plate at a temperature between 550 to 600 °C and subsequently quenched in the DI water for its shape setting following a standard protocol^15^. In the post-heat treatment, the SMA wire fixture was removed from the steel wire base by unwinding the copper wire and soldered with another set of copper wire for the electrical current transmission.

The mould for artificial cilia and microchannel with a side chamber for SMA wire fixture was carved onto an acrylic substrate of thickness 5 mm. The microchannel fabrication flow layout is shown in the Fig. 1(c). The geometric pattern for the microchannel with moving wall structure was engraved through several steps of micromachining operation. To fabricate the artificial cilia, micro-holes with a diameter of 250 μm having a depth of 1.5 mm were drilled on the mould of the microchannel surface through micro-drilling operation where artificial cilia elastomer introduced subsequently. This elastomer is a homogeneous and degassed mixture of commercially available neodymium-iron-boron magnetic particles of 5 μm diameter (MQP-15-7, Magnequench, Singapore) and PDMS (PDMS, Sylgard 184, Dow Corning Corp., Midland, MI, USA) in a weight ratio of 4:1. Thereupon a mixture of PDMS base and curing agent in a weight ratio of 10:1 were degassed by using a vacuum pump, subsequently poured on the desired mould carved for the microchannel. Then mould and microchannel replica had undergone soft curing process at temperature of 95 °C for 48 hours and microchannel replica was removed from the parent mould. Once the microchannel replica was removed, holes were drilled for inlet and outlet tubing for the introduction of zebrafish larvae, and microchannel openings were sealed by glass coverslip through oxygen enriched plasma treatment process. Heat-treated SMA wire fixture was further returned in straight, and placed into the side chamber of the microchannel in parallel to the wall of the microchannel (Fig. 1d). Deformation of the microchannel wall were then achieved by varying temperature of the SMA wire fixture (by means of varying the electrical current input) so that it can attune its original arc shape and provide the desired displacement to the microchannel wall.

### Control system for artificial cilia and moving wall

Towards motion control of artificial cilia, an external magnetic coil system was employed. The cores of the magnetic coil system were made-up of soft iron cores by winding the insulated copper wire of diameter of 0.25 mm. Through this system a homogeneous electro-magnetic field was generated and artificial cilia motion was controlled. Desired power for the coil system was controlled through a power supply unit (GPR-3510HD DC Power Supply, Instek, Taiwan) via the input and output modules of the data acquisition device (NI cDAQ-9174, National Instruments, Austin, TX, USA). A customized GUI was developed towards implementing the control algorithm through an in-house script. The detailed description of the algorithm and beating patterns of artificial cilia can be found elsewhere^13^.

**Figure 2:**
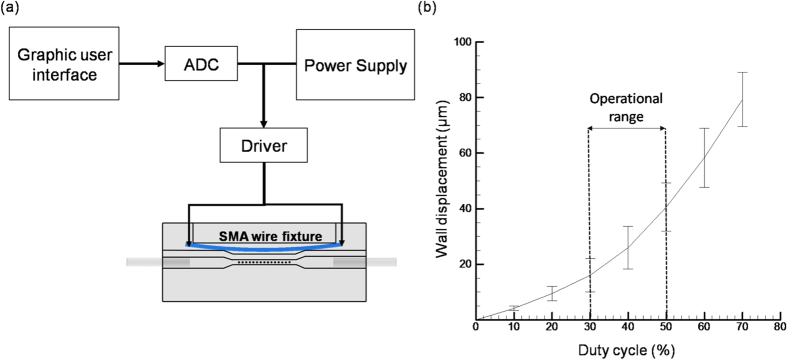
(**a**) Schematic illustration showing the electrical control system for the moving wall structure of the microchannel through SMA wire fixture. (**b**) The relationship between the displacements of the moving wall corresponding to the increase in duty cycle.

Additionally, for the control of moving wall structure of the microchannel through SMA wire fixture, an electrical control system was deployed. As illustrated in the Fig. 2(a), the control system consists a tailor made GUI to implement the algorithm, ADC converter (Arduino Leonardo R3, Arduino, Italy), driver and a power supply system (Regulated DC power supply, Chern Taih Corporation, Taiwan). In order to provide accurate control of SMA wire, the current was supplied by virtue of PWM (pulse width modulation) waveform through the driver which allows the exact control of electrical current for heat generation distributed evenly throughout the SMA fixture.In order to quantify the strength of the generated electric field towards the deformation the microchannel wall, the direct link between the displacements of the wall corresponding to the increase in the duty cycle of the PWM waveform was investigated. The deforming wall area can be defined as the gap between the artificial cilia and the microchannel wall. As shown Fig. 2(b) a higher deformation of walls (corresponding to the original wall position priori to application of electrical current) can be achieved with the increase in duty cycle. The duty cycle is defined as the percentage of the signal stays active compared to the resting state. For example, a duty cycle of 70% refers to the signal is on during 70% of the total cycle time but it is off in the rest 30% cycle time. The higher the duty cycle denotes the longer time the signal stays active which enables more electrical current to pass through the SMA fixture for stronger wall deformation. Moreover, a maximum deformation of 80 *μ*m can be achieved at a duty cycle of 80% for the above mentioned microchannel dimensions. However, the operational range of the duty cycle was determined from 30 to 50 as it fits the overall morphology of the zebrafish larvae considered in this study.

### Quantification of axial rotation through *in-vitro* validation

**Figure 3:**
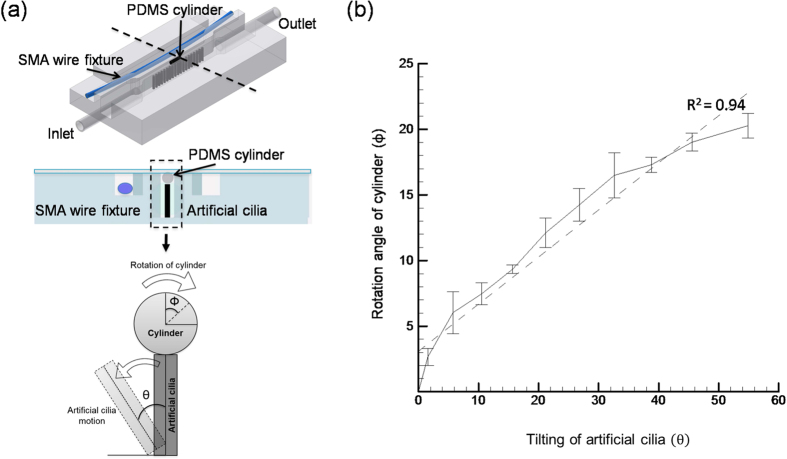
(**a**) The top two figure of the left panel explains the relative position of PDMS cylinder on the artificial cilia, the bottom figure of the left panel explains the mechanism of the cylinder’s rotation corresponding the artificial cilia tilting angle. (**b**) The relative rotation of cylinder corresponds to the artificial cilia tilting is quantified. *Dashed line* represents the linear fitting to resultant curve, where *R*^*2*^ > 0.9. The error bar shows one standard deviation over three measurements.

In order to demonstrate and pre-validate the stepwise rotation of zebrafish larvae in an axial manner through artificial cilia actuation, an experiment was carried out with a PDMS cylinder that mimics the shape of the zebrafish as shown in Fig. 3(a). The cylinder has a diameter of 0.505 mm and a length of 1.25 mm, fabricated using negative mould technique and introduced in the microchannel through inlet tubing. The cylinder was aligned and centered in the microchannel by applying negative pressure through the output tubing. The stepwise axial rotation of the cylinder was performed by the virtue of direct contact between cylinder and artificial cilia through the titling action (with respective to the microchannel bottom wall) of artificial cilia. The relation between the relative axial rotations of cylinder 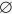
corresponding to the tilting angle of artificial cilia 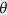 was quantified by plotting them in the Fig. 3(b). Specifically, this rotational angle of the cylinder was quantified by calibrating the change in length between two selected points on the tested cylinder by projecting them on the imaging plane during each step of their rotation. In details, during each step of artificial cilia tilting, images along the plane with the cylinder placed on top of the artificial cilia were recorded through the aforementioned high speed camera and an open source image tracking software: “DLT Dataviewer^16^” was used to track the projected changes of length between two specific points. In a similar manner, the tilting angle of artificial cilia was quantified by tracking the projected change of a premeditated point on the edge of artificial cilia and the initial position of the cilia center in the imaging plane. A series of image preprocessing techniques such as contrast enhancement and noise reduction were employed prior to the calculation of projected length changes in the respective distance of the cylinder points and artificial cilia tip. By means of the presented actuation method both the clockwise and counterclockwise rotations of the cylinder can be achieved by tilting the artificial cilia in an opposite manner towards the desired rotation of interest. The experiment findings reveal that a substantial rotation of 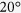 can be achieved corresponding to a 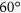 tilting of artificial cilia (Fig. 3b) in a stepwise manner with a minimal axial rotation resolution of 2-degree. The higher coefficient of determination (*R*^*2*^ > 0.90) manifests the linear relationship between both the angles and explains that the step wise rotation of cylinder is unbiased and uniform by nature. In details a robust stepwise rotation of cylinder can be achieved corresponding to the small increment in artificial cilia tilting angle. However, this higher degree of uniformity in the rotation was due to the symmetric structure of the cylinder hypothesized to be different for zebrafish as it possesses an asymmetric morphology and might possesses a different behavior. Therefore, in this work the moving wall design was integrated into the artificial cilia-based microchannel for the orientation control of zebrafish which is capable of adapting repaid morphological changes of zebrafish under developing.

### Quantification of axial rotation through *in-vivo* investigation

**Figure 4:**
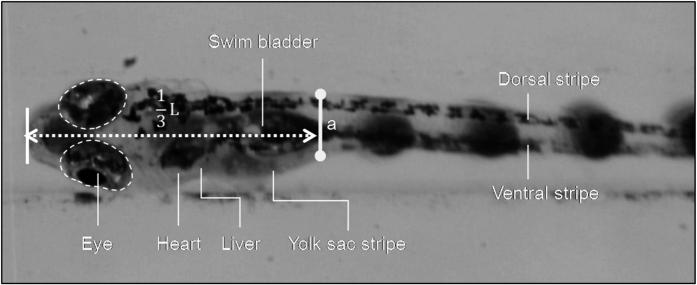
Photograph of a 5 d.p.f. zebrafish illustrating the important organs and the measuring location. ‘L’ denotes the total length of the tested zebrafish. A projected length ‘a’ was measured to provide quantitative results of zebrafish axial rotation.

To demonstrate the rotational capability of the zebrafish through the proposed artificial cilia-based microchannel, a definitive position which is situated approximately 1/3^rd^ of the zebrafish length from the anterior end was marked (Fig. 4) throughout the experiment. Irrespective of the zebrafish age, this method was adopted so that uniformity and consistency in the results can be achieved. The rotational angle of the zebrafish 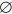 and tilting angle of the artificial cilia 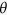 was measured in a similar manner as in the method used to calibrate the rotation of cylinder. A series of experiments were conducted with and without wall activation to demonstrate the efficacy of the proposed device towards accommodating and rotating zebrafish of different age groups (i.e. 1 d.p.f to 6 d.p.f.) through moderate stepwise increment in the artificial cilia tilting. Specially, in the test cases for zebrafish from 1 d.p.f. to 3 d.p.f., no moving wall structure was activated. However, in the cases of zebrafish from 4 d.p.f. to 6 d.p.f., results with and without moving wall activation were documented. The corresponding wall displacement values in the moving wall activation cases were 15 μm, 25 μm, and 40 μm for zebrafish at 4 d.p.f., 5 d.p.f., and 6 d.p.f., respectively.

### Statistical analysis

In order to examine the effect of wall activation on the repeatability of the embryo rotation, the standard deviation of the embryo rotation angle was computed for each of the examined artificial cilia tilt angles. Each artificial cilia tilt angle was treated as an independent sample, and the standard deviation with and without wall activation were compared using a paired, two-tailed t-test (N = 11).

## Results and Discussion

**Figure 5:**
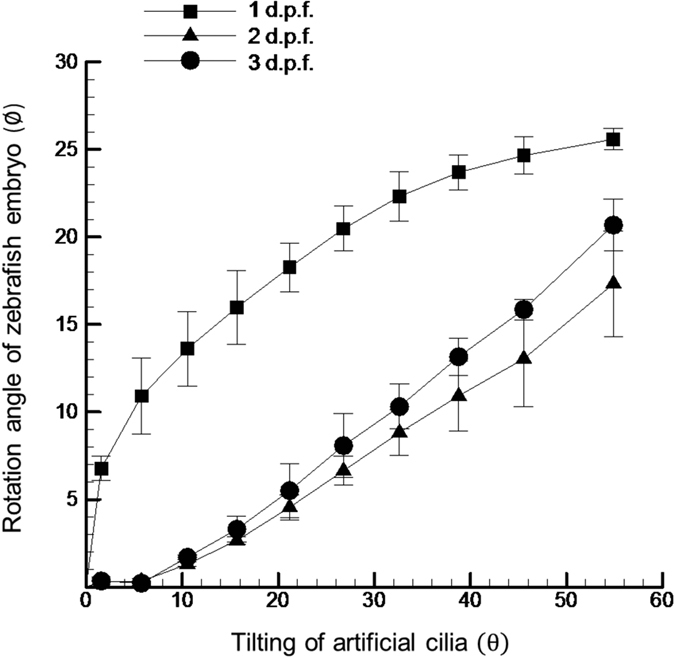
Quantification of the relative rotation of the early embryonic zebrafish (1 to 3 d.p.f.) corresponding to the artificial cilia tilting. *R*^*2*^ of these three curves was found to be greater than 0.85. Each zebrafish is tested for three times with three zebrafishes tested in total. Error bars denote one standard deviation over nine measurements.

Through this proposed platform, zebrafish at earlier age groups (up to 9 d.p.f.) can be rotated in a controlled manner through the actuation of artificial cilia. The relative axial rotation of the zebrafish corresponding to the tilting action of artificial cilia for zebrafish aging from 1 d.p.f to 3 d.p.f. was measured with results provided in Fig. 5. As illustrated, this animal model in its early embryonic stages achieves an average axial rotation of 20 degrees when a substantial rotational angle of artificial cilia of almost 60 degrees was achieved through the in-house magnetic coil system. The test results with the axial rotation of zebrafish are thoroughly consistent with the test results of cylinder provided in a previous section, disagrees with the prediction of hypothesis where no significantly asymmetric morphology of zebrafish was found at these development stages. More importantly, the stepwise rotational movement of the early zebrafish can be controlled precisely through moderate tilting of artificial cilia. These findings are affirmed from the higher degree of linearity (*R*^*2*^ > 0.85) of the resultant curve fitting between the rotational motion of the zebrafish and artificial cilia tilting. Still, the curve trend of 1 d.p.f. data differs from the other two groups where a rapid increase was found initially from the curve and the response of the zebrafish axial rotation became less sensitive to the relative large tilting of artificial cilia thereafter. A possible speculation is given to illustrate this observation as for 1 d.p.f. zebrafish its body is more flexible compared to zebrafish at later stages which leads to a larger contact area between the zebrafish and artificial cilia under the same applied contact force. As a result, the response of zebrafish to perform axial rotation is more or less hindered, and the less degree of controllability to the zebrafish manipulation was obtained.

**Figure 6:**
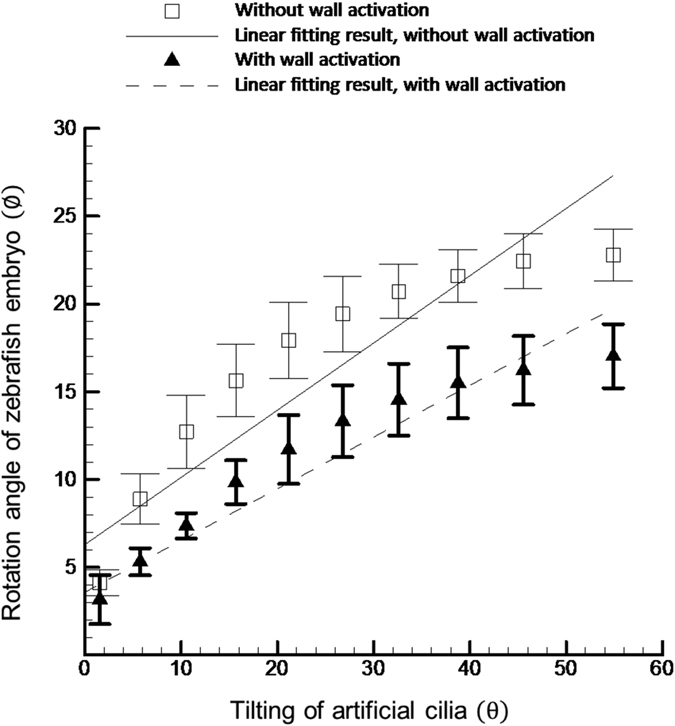
Quantification of the relative motion of 4 d.p.f. zebrafish embryo corresponding to the artificial cilia tilting with and without wall activation. The solid and dotted line represents the linear fitting results of the measured curves. A higher degree of linearity (*R*^*2*^ = 0.89) was found with wall activation compared to that without wall activation (*R*^*2*^ = 0.81).

**Figure 7:**
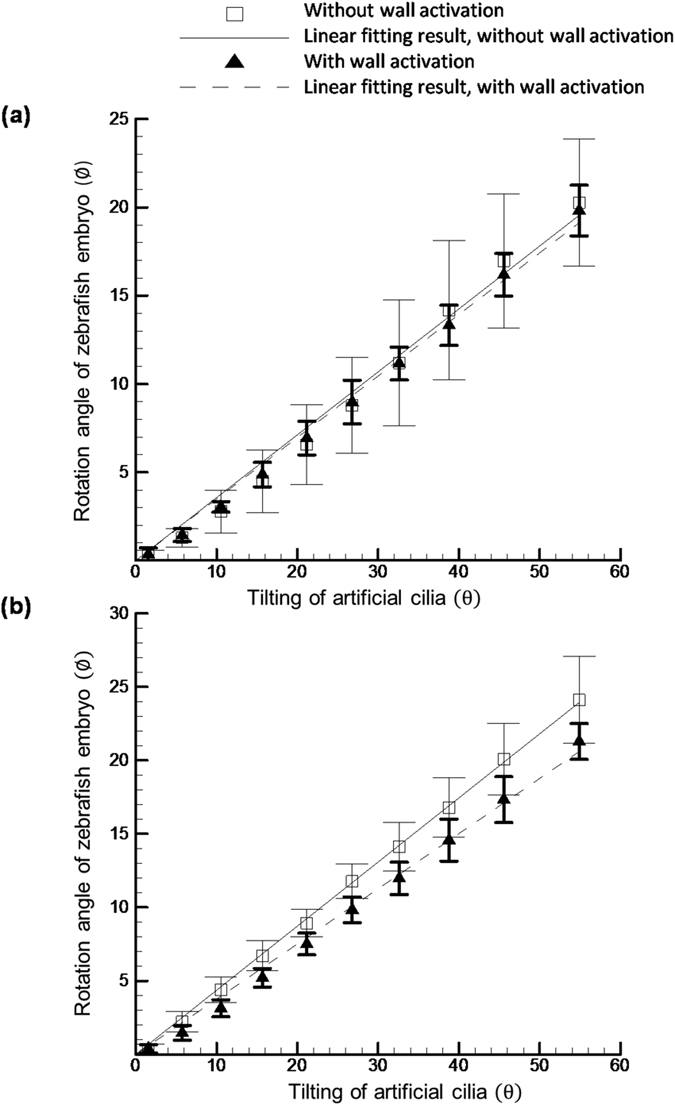
Quantification of the relative rotation of 5 d.p.f. (**a**) and 6 d.p.f. (**b**) zebrafish corresponding to the artificial cilia tilting with and without wall activation. The solid and dotted line represents the linear fitting results of the measured curves.

With the advent of age (i.e. >3 d.p.f.), the width of the zebrafish decreases on an average of 15 μm per each day (data not shown) as the yoke shrinks and body elongates towards the tail. Hence, the moving wall structure of the microchannel was activated so that zebrafish can be precisely entrapped inside the microchannel and the stepwise rotation can be performed with an ease. To demonstrate the efficacy of the integration of the moving wall structure with the artificial cilia based microchannel, the experiment was conducted with and without activating the moving wall structure. Three different specimens of each age group (4–6 d.p.f.) were considered for the study. The relative rotation of the zebrafish corresponds to the tilting angle of artificial cilia was quantified and graphically represented in the Figs 6 and 7. In Fig. 6, for zebrafish at 4 d.p.f., it was observed that a higher degree of linearity between the rotational angle of the zebrafish and tilting angle of artificial cilia was evidenced when the experiment was carried out with the moving wall activation (*R*^*2*^ = 0.89) than that without moving wall activation (*R*^*2*^ = 0.81). This finding provides a solid evidence on the need to implement the moving wall design to zebrafish orientation control. Still, there is a certain decrease in the magnitude of the rotational angle of zebrafish corresponding to the maximum tilting of artificial cilia, when the moving wall structure was activated. For instance, an average rotation of 15 degrees was achieved for a 4 d.p.f. zebrafish when the moving wall structure was activated, compared to an angle of 20 degrees when the moving wall was not activated. However, this issue can be resolved by increasing the duty cycle level of the coil system to reach a higher titling angle of artificial cilia which lead to the larger rotation angle of zebrafish (data not shown). In short, the efficacy of the proposed moving wall concept was demonstrated which is envisaged that is beneficial for that will further facilitates the time lapse imaging of the specimen.

Considering the morphology and size of zebrafish differ significantly with each other even at the same age group, it has been always a challenge to control their orientation inside the microchannel accurately. As shown in the Fig. 7, for 5 and 6 d.p.f. zebrafish tested results, the measured deviations in the rotational angles were observed when the experiment was carried through the with and without activation of moving wall. However, the deviation is relative larger in the without moving wall actuation group than that with the moving wall activation group, as evidenced in the following reported statistic values. For instance, the average standard deviation value for the zebrafish on 5 d.p.f. in the without moving wall activation group was estimated as 2.15-degrees whereas this value decreased to 0.78-degree in the group with moving wall activation, which is statistically significant (*p* < 0.05). This might be originated from the effect of the significant zebrafish morphological change which leads to the gap between the zebrafish and the microchannel wall arises. The enlargement of this gap hinders the accurate control of zebrafish orientation since the tested zebrafish may drift inside the microchannel due to the increment of the gap. Moving one step further to collect the results for zebrafish on 6 d.p.f., consistent behaviors were identified. The statistics shows that the average standard deviation was 1.28-degree in the case of without moving wall activation but the value dropped to 0.81-degree in the with moving wall activation group, which is statistically significant (*p* < 0.05). Looking at the objective of this work where the stepwise rotation in a range of 0 to 20 degrees is desired to acute orientation control of zebrafish in time-lapse imaging, this downturn of the standard deviation value can be beneficial especially for the high magnification imaging where slight movement of sample may degrade the imaging quality significantly. With this improvement by reducing the deviation during orientation control of zebrafish such negative effect can be reduced to a minimal level. These findings illustrate the significance of the moving wall structure towards the orientation control of zebrafish in genetic screening.

Moreover a single microfluidic platform is unavailable till date which can facilitate and accommodate the rapid growth of the vertebrae model throughout their embryonic developmental phases. The aforementioned statement can be verified in a recent manuscript^17^ where the authors have reviewed zebrafish manipulation platforms available till date and listed specifications of each devices. Our proposed microfluidic platform with a moving wall function will be able to advent the zebrafish entrapping technologies in terms of accommodating the vertebrae model throughout their embryonic developmental stages. Through the proposed system, the matter of interest can be entrapped and rotated precisely in a stepwise manner through artificial cilia actuation for time-lapse imaging in order to interpret the hemodynamics during development for studying its cardiac anomalies as well as drug and genetic screening. As mentioned earlier, this proposed platform will be also beneficial for the 3D reconstruction of cardiovascular networks for species in development that can further improve the current understanding of angiogenesis and vasculogenesis. In order to demonstrate the step wise time-lapse imaging capability of our proposed device, 3D reconstruction of the cardiovascular network of a 4 d.p.f zebrafish embryo was carried out and illustrated in the Supplementary Material. This manipulation concept not only can be applied to study zebrafish as demonstrated in this work but also can be extended to other tiny animal models such as C. elegans which is also a popular animal model for biomedical investigations after some minor modifications of the presented device.

## Conclusions

An artificial cilia based microchannel with moving wall capability through SMA material actuation was reported in this work with a specific function to accommodate zebrafish throughout the major early developing stages for facilitating time-lapse imaging in genetic screening. The reported moving wall design is capable of deflecting the microchannel wall up to 40 μm which enables zebrafish imaging from 1 d.p.f. to 6 d.p.f. with a promising extension to later stages. Compared with the case of no moving wall actuation, by means of the reported moving wall feature a better control of zebrafish orientation was obtained. This is evidenced by a higher degree of linearity of rotational angle relation between the artificial cilia array and zebrafish was observed in conjunction with less angle deviations when multiple zebrafishes were tested. The presented results advance the current zebrafish imaging process by introducing the moving wall concept into the zebrafish manipulation which is beneficial to contemporary gene screening using animal models.

### Additional Information

This study was supported through Ministry of Science and Technology of Taiwan under Contract No. MOST 104-2221-E-006-169 and No. MOST 102-2221-E-006-297-MY3 (to Chia-Yuan Chen). This work would not be possible without the facility provided by Center for Micro/Nano Science and Technology, National Cheng Kung University. The research was in part supported by the Minsitry of Education, Taiwan, R. O. C. through the Aim for the Top University Project to National Cheng Kung University (NCKU).

## Additional Information

**How to cite this article**: Mani, K. *et al*. Manipulation of zebrafish’s orientation using artificial cilia in a microchannel with actively adaptive wall design. *Sci. Rep*. **6**, 36385; doi: 10.1038/srep36385 (2016).

**Publisher’s note:** Springer Nature remains neutral with regard to jurisdictional claims in published maps and institutional affiliations.

## Supplementary Material

Supplementary Information

Supplementary Video S1
