# Supplementary material for: Manipulation of zebrafish’s orientation using artificial cilia in a microchannel with actively adaptive wall design
Source: Sci Rep. 2016 Nov 8;6:36385. doi: 10.1038/srep36385 (PMC5099576; doi:10.1038/srep36385)
Supplement: Supplementary Information [file srep36385-s1.doc]

**Supplementary information**

**Manipulation of zebrafish's orientation using artificial cilia in a microchannel with actively adaptive wall design**

Karthick Mani^1^, Tsung-Chun Chang Chien^1^, Bivas Panigrahi^1^,

and Chia-Yuan Chen^1＊^

^1^Department of Mechanical Engineering, National Cheng Kung University, Tainan 701, Taiwan

Address for Correspondence:

Chia-Yuan Chen, PhD.

3D reconstruction of cardiovascular network for zebrafish:

This proposed platform is beneficial for 3D reconstruction of cardiovascular networks in development which can improve the current understanding of angiogenesis and vasculogenesis. To demonstrate this statement, a quick experiment was conducted where a zebrafish of 4 d.p.f. was imaged during the axial rotation with the help of this presented platform. Thereafter, a series of image processing algorithms was employed to reconstruct the cardiovascular networks from the images of the zebrafish acquired at different rotational angles. Specifically, a series of 20 images was captured at different focal depths, where each image focused to a particular plane (in-focus of the zebrafish). In-focus regions of each image were detected automatically and stacked together as single image. This process was repeated while axially rotating the zebrafish so that images can be captured at different regions of the zebrafish. 3D reconstruction of the cardiovascular network from the pre-processed images was performed. In this step the region of interest was selected and highlighted together with the assembling of all captured images. The cardiovascular system was therefore reconstructed. Figure S1. and S2 illustrates the 3D vascular network consisting of heart and peripheral blood vessels of the 4 d.p.f zebrafish.


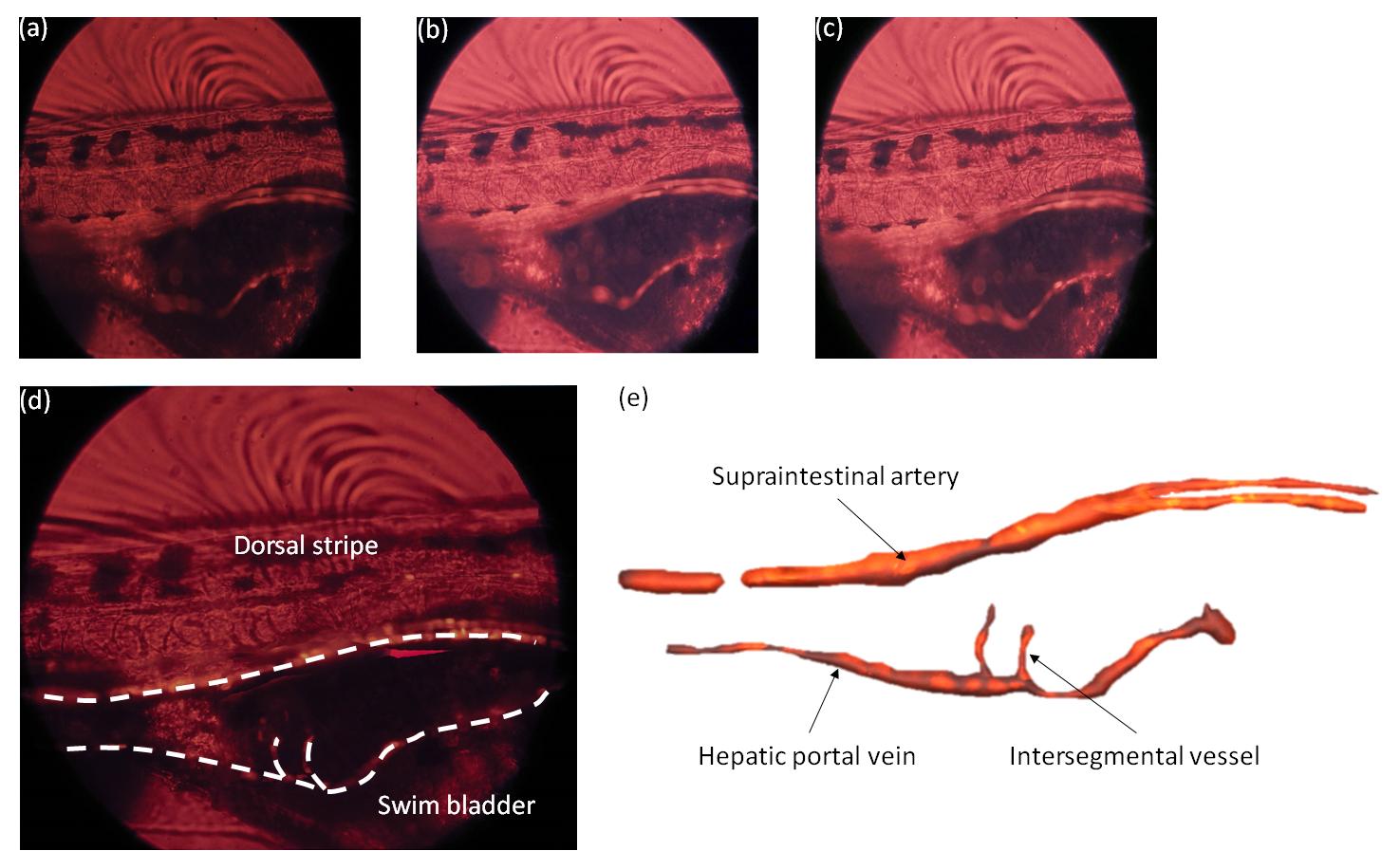


Figure S1. 3D reconstruction of selected vessels of interest through the present zebrafish orientation manipulation platform. Sampled images of 4 d.p.f. zebrafish were captured at various axial rotational angles with respect to the original position through the modulation of duty cycles from 0% to 100% with 10% increment. Representative images at duty cycles of 10% (a), 20% (b), 30% (c), and 40% (d). (e) Final 3D reconstructed vascular network.


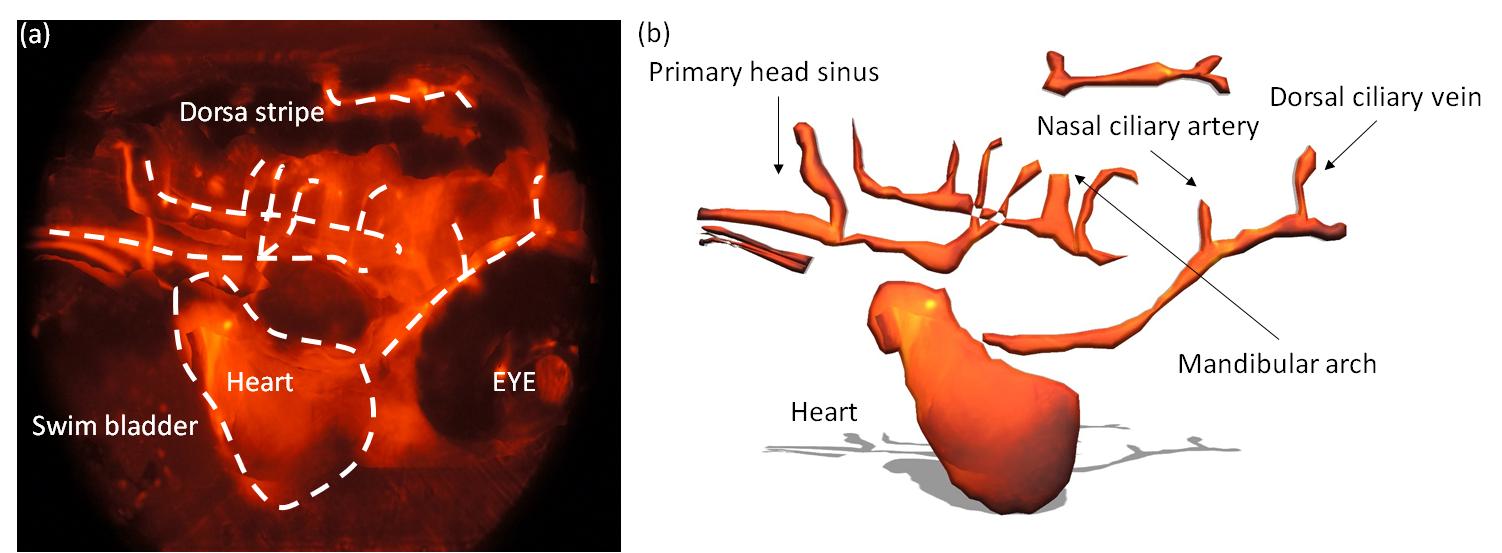


Figure S2. 3D reconstruction of heart and peripheral vascular network through the present zebrafish orientation manipulation platform. The tested zebrafish is at the development stage of 4 d.p.f. Captured 2D and reconstructed results are shown in panels (a) and (b), respectively.

**Supplementary Video 1.** Detailed depiction of the microchannel with moving wall structure. To demonstrate the capability of the microfluidic platform, a 5 d.p.f. zebrafish larva was rotated in an axial manner through the artificial cilia actuation together with SMA-actuated moving wall feature.
